# Effects of learning content in context on knowledge acquisition and recall: a pretest-posttest control group design

**DOI:** 10.1186/s12909-015-0416-0

**Published:** 2015-08-15

**Authors:** Esther M. Bergman, Anique B. H. de Bruin, Marc A. T. M. Vorstenbosch, Jan G. M. Kooloos, Ghita C. W. M. Puts, Jimmie Leppink, Albert J. J. A. Scherpbier, Cees P. M. van der Vleuten

**Affiliations:** 1 Department of Educational Development and Research, Faculty of Health, Medicine and Life Sciences, Maastricht University, P.O. Box 616, 6200 MD Maastricht, The Netherlands; 2 Department of Anatomy, Radboud University Medical Centre Nijmegen, Nijmegen, The Netherlands; 3 Radboud University Medical Centre Nijmegen, Nijmegen, The Netherlands; 4 Faculty of Health, Medicine and Life Sciences, Maastricht University, Maastricht, The Netherlands

## Abstract

**Background:**

It is generally assumed that learning in context increases performance. This study investigates the relationship between the characteristics of a paper-patient context (relevance and familiarity), the mechanisms through which the cognitive dimension of context could improve learning (activation of prior knowledge, elaboration and increasing retrieval cues), and test performance.

**Methods:**

A total of 145 medical students completed a pretest of 40 questions, of which half were with a patient vignette. One week later, they studied musculoskeletal anatomy in the dissection room without a paper-patient context (control group) or with (ir)relevant-(un)familiar context (experimental groups), and completed a cognitive load scale. Following a short delay, the students completed a posttest.

**Results:**

Surprisingly, our results show that students who studied in context did not perform better than students who studied without context. This finding may be explained by an interaction of the participants’ expertise level, the nature of anatomical knowledge and students’ approaches to learning. A relevant-familiar context only reduced the negative effect of learning the content in context. Our results suggest discouraging the introduction of an uncommon disease to illustrate a basic science concept. Higher self-perceived learning scores predict higher performance. Interestingly, students performed significantly better on the questions with context in both tests, possibly due to a ‘framing effect’.

**Conclusions:**

Since studies focusing on the physical and affective dimensions of context have also failed to find a positive influence of learning in a clinically relevant context, further research seems necessary to refine our theories around the role of context in learning.

**Electronic supplementary material:**

The online version of this article (doi:10.1186/s12909-015-0416-0) contains supplementary material, which is available to authorized users.

## Background

The impact of context is of particular interest in medical education, since students commonly learn basic science knowledge in the medical school and apply it in the clinical workplace [1, 2]. It is generally assumed that learning in context will increase performance in knowledge acquisition, recall and transfer [3, 4]. However, studies with a sound research design determining the effect of teaching in context on performance are scarce [5]. Paper-patient cases (patient vignettes) are used as a context to learn e.g. diagnostic skills [6] and professional behavior [7]. This study aims to contribute to the literature on learning in context by investigating whether and how a paper-patient case used as context in basic science education can influence the acquisition and recall of knowledge.

Leading up to our research questions, and as explanation for our research design, we will first discuss the theories of: dimensions of context, semantic knowledge networks and cognitive load. These theories underpin our ideas about the possible influence of a paper-patient context on learning and retrieval.

## Dimensions of context

**Fig. 1:**
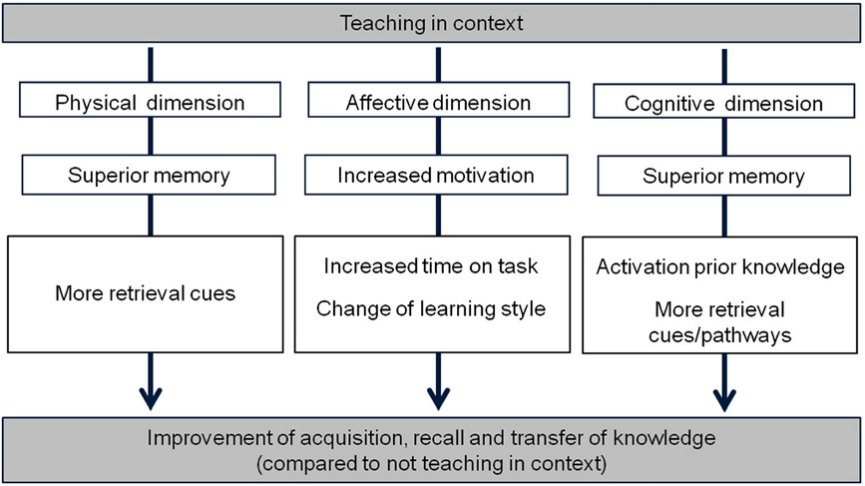
Dimensions of context model (adapted from Koens et al. [9])

Koens et al. (2005) developed a model with three ‘dimensions of context’ (see Fig. 1): the physical dimension, the affective (commitment) dimension and the cognitive (semantic) dimension.

The physical dimension refers to the physical surroundings in which the content is presented to the learner. Extensive research on the physical dimension (for a review see Smith and Vela [8]), has pointed towards a ‘same-context advantage’, explained by the idea that cues present in the environment during learning are encoded in the memory along with the content. When these cues reappear in the environment in which the content is recalled, they facilitate retrieval of the content [9].

The affective dimension focuses on the influence of context on the learner’s willingness to invest effort in (motivation for) the learning task. The affective dimension is stated to manifest itself in two ways: increased ‘time on task’ and/or a shift in learning style from superficial to more deep learning. Both can lead to an increase in the acquisition, understanding and retention of knowledge [9].

Providing a paper-patient case as a context to learn basic science knowledge (as is for example done in problem-based learning [10, 11]), appeals to the mechanisms through which the *cognitive dimension* of context should improve learning and retrieval: activation of prior knowledge, storage of retrieval cues and elaboration (storage of retrieval pathways). Our research design is developed to study the effects of a paper-patient case on these mechanisms, but before we can explain this further, it is important to describe how the learned content is thought to be stored in memory.

## Characteristics of context and semantic networks

Information is thought to be stored in memory by the creation of knowledge structures called *semantic networks*. Semantic networks consist of one or more *propositions*, which are described as statements that contain information parts (concepts, facts, experiences) and their meaningful interrelations or ‘links’ [12, 13]. Knowledge is thought to be retrieved through activation of a semantic network [12], and the structure of a semantic network influences its accessibility [13–15].

Semantic networks are idiosyncratic: they reflect a person’s experiences, views and ideas and therefore no two persons have the same knowledge about a certain topic [13, 16]. The quality of a semantic network, i.e. the detail, amount, accuracy and organization of propositions, may be influenced by presenting the content in context. In addition, specific characteristics of that context, in this study defined as relevance and familiarity, may influence the quality of a semantic network even further.

### Influence of the relevance of context

As described above, one of the mechanisms through which the cognitive dimension of context should improve *retrieval* is by enhancing the storage of retrieval cues. Similar as the encoding of environmental cues that explains the same-context advantage of the physical dimension, cues of the paper-patient context may be stored along with the content as information parts in the propositions of a semantic network. As each proposition can be a means to recall all the other knowledge stored in a semantic network, learning in a paper-patient context can consequently facilitate retrieval of the content by increasing the number of retrieval cues.

The other mechanism through which the cognitive dimension of context should improve *retrieval* is through elaboration; by increasing the amount of retrieval pathways. Elaboration means that the learner generates meaningful connections between information parts (either from prior knowledge, the new context and/or content), thus integrating new information within an existing semantic network [13, 14, 17]. The paper-patient context gives the opportunity to create more links between information parts, thus enriching a students’ semantic network [18].

Interestingly, “it is important to note that the context includes all features of the environment at the time of learning, not simply those judged by some external criterion to be, in some way, important or relevant to the material learned” [19]. One characteristic of the context is relevance, in this study defined as the extent to which the disease of the paper-patient case is associated with content to be learned. To increase the amount of retrieval cues/pathways, the given context should be relevant to the content to be learned; otherwise the creation of propositions will be limited as making meaningful connections is hindered. Obviously, providing students with a paper-patient case irrelevant to the to-be-learned content is not something which would be applied in educational practice. However, it is possible that an irrelevant context may increase the amount of retrieval cues and retrieval pathways compared to learning without context, although not as much as a relevant context. Because we also aim to contribute to the literature on learning in context, this study investigates the influences of all the different conditions.

### Influence of the familiarity of the context

The mechanism through which the cognitive dimension of context should improve *learning* is the activation of prior knowledge. Schmidt [13] states that “the prior knowledge people have regarding a subject is the most important determinant of the nature and amount of new information that can be processed”. However, the mere availability of relevant prior knowledge is not sufficient: prior knowledge needs to be activated before links between prior knowledge and new information can be made [13]. The paper-patient context may stimulate prior knowledge activation by triggering the retrieval of information about the content that a student has already stored in a semantic network. This activation of prior knowledge might not happen if the content was presented without context.

Another characteristic of the context defined in this study is ‘familiarity’, in this study defined as the extent to which the students are familiar with the disease of the paper-patient. As semantic networks are idiosyncratic, a presumably familiar context (i.e., a paper-patient with a common disease) increases the chance of the presence and activation of an appropriate semantic network in students. A familiar context may therefore improve learning further than an unfamiliar disease, as the latter triggers less prior knowledge.

Although the interaction between the characteristics of context and the mechanisms through which the cognitive dimension of context should improve learning as described above may seem obvious, they are hypothetical. To our knowledge, there is no empirical evidence to support these ideas.

## Context and cognitive load

It is possible that learning in context is not always beneficial, because it increases cognitive load. The central tenet of cognitive load theory is that human cognitive architecture – and especially the limitations of working memory – should be taken into account when designing instruction [20]. Working memory has a limited capacity of seven plus or minus two elements (or chunks) of information when merely holding information [21] and even fewer (circa four) when processing information [22]. Working memory load, or cognitive load, is therefore determined by the number of information elements that need to be processed simultaneously within a certain amount of time [23]. Proper measurement of cognitive load can help us understand why the effectiveness and efficiency of learning environments may differ as a function of instructional formats and learner characteristics.

In the traditional cognitive load theory framework [24, 25], three types of cognitive load may be imposed on a learner’s working memory when processing (complex) information: the complexity of the content or task imposes an intrinsic cognitive load depending on the learner’s expertise or prior knowledge of the subject; instructional or context features that are unnecessary or inappropriate for learning contribute to extraneous cognitive load; and instructional features that contribute to learning determine the germane cognitive load. Recently, a ten-item instrument was developed that aimed to measure these three types of cognitive load [26, 27]. However, in a more recent framework, germane cognitive load is no longer perceived as a third type of cognitive load but as that part of intrinsic cognitive load that actually contributes to learning [27, 28]. In line with this development and a lack of empirical evidence for germane cognitive load measurement [27], the latter factor is currently interpreted as self-perceived learning. We will use this interpretation during the remainder of this study.

For the last three decades it has been advocated that medical education should be integrated, or in other words, its content should be presented in a context that is relevant for the medical professional. Next to reducing the ‘shock of practice’ [29] and helping to focus on the clinically relevant aspects of the basic sciences [30], teaching in context is said to contribute to knowledge acquisition and retrieval as described above. This study aims to contribute to the literature on teaching in context by investigating whether and how a paper-patient used as a context during learning of a basic science can influence acquisition and recall of knowledge. Following the literature and theories described above we generated four hypotheses:

1. Learning with a paper-patient context leads to better performance than learning without context
2. A relevant paper-patient context leads to better performance than irrelevant context
3. A familiar paper-patient context leads to better performance than unfamiliar context
4. Higher scores on self-perceived learning predict higher performance

## Methods

Ethical approval for this study was obtained from the Dutch Association of Medical Education (NVMO) ethical review board. The students enrolled voluntary in this study and a written informed consent was obtained from all the participants. The students received a dinner on day 2 and a small financial fee for their participation.

### Setting

This study was conducted at the Radboud University Medical Centre Nijmegen which has a 6-year problem-oriented, student-centered, integrated curriculum.

### Participants and experimental design

A total of 145 first-year (bachelor) medical students (see Table 1 for descriptives) were allocated randomly to one of five experimental treatment conditions: (1) the control group taught without context, and the experimental groups respectively being taught with (2) relevant-familiar, (3) relevant-unfamiliar, (4) irrelevant-familiar, and (5) irrelevant-unfamiliar context (see also the ‘procedure’ section).

**Table 1:** Descriptives of participants

Age	Average 19,2 years
	Range 17.5-23.6 years
Gender	112 female
	33 male
Nationality	138 Dutch
	3 German
	1 Yugoslav
	1 Iraqi
	1 Turkish
	1 Bulgarian
Relevant previous education	15 (4 Biology, 4 Biomedical sciences/technology, 1 Technical Medicine, 1 Psychology, 1 Health sciences, 1 Pharmacology, 3 other)
	All students quit their other education upon acceptance into medical school

**Fig. 2:**
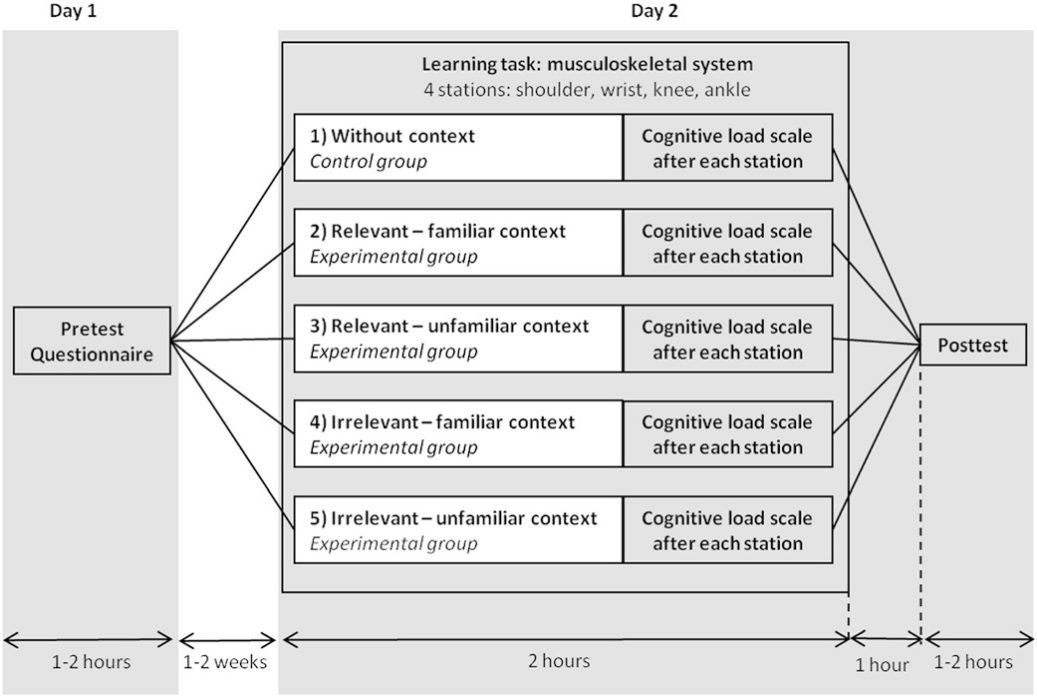
Experimental design. On day 1, students completed a pretest assessing their knowledge about musculoskeletal anatomy and a questionnaire assessing their familiarity with the diseases used in the paper-patient context. On day 2, students completed a learning task in the dissection room rotating through four different stations. After each station they completed a cognitive load scale. One hour after treatment, all students completed the post-test

The flowchart of the experiment is depicted in Fig. 2. Students completed a pre-test before and post-test after treatment. Both pre-test and post-test consisted of 40 extended matching questions about the four musculoskeletal regions that were studied in the learning task (10 questions each). Half of the questions were with and half of the questions were without context (patient vignettes). Students did not receive feedback on their test performance.

The students completed a questionnaire in which they could indicate their familiarity with the disease on which the paper-patient case (the context) in the learning task was based. The options were: 0 for ‘I have never heard of this disease’, and then a 5 point Likert scale with 1 being ‘I have little knowledge of this disease’ until 5 being ‘I have a lot of knowledge of this disease’.

### Procedure

**Fig. 3:**
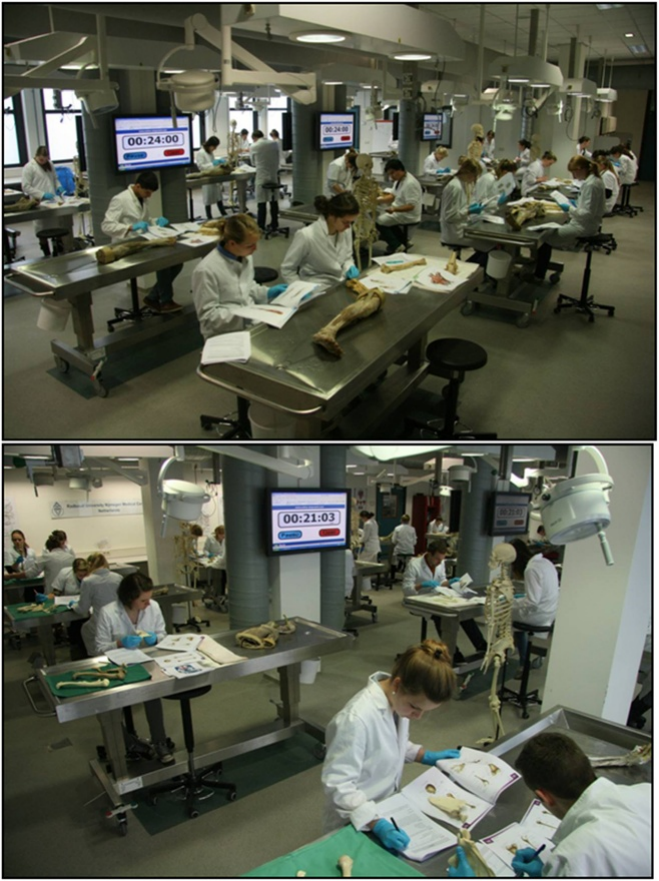
Students are studying four musculoskeletal regions (shoulder, wrist, knee, ankle) in the dissection room using skeletal material, prosected parts of cadavers, copies of pages of an anatomical atlas and textbook, and a manual especially written for the experiment containing the context ((ir) relevant and (un) familiar patient case) and the to-be-learned anatomical content

The learning task was 2 h structured learning session in the dissection room [31]. The planning of these learning sessions was purposefully chosen to stimulate students to participate in the experiment (i.e. they were scheduled after mandatory lessons in the regular curriculum). Students rotated in counterbalanced order through 4 different stations focusing on different musculoskeletal regions: shoulder, wrist, knee and ankle (Fig. 3). A student manual was written containing the paper-patient case followed by questions and assignments around relevant structures of the regions (see Additional file 1). At each station, students had 25 min to study the content using the provided anatomical material. The remaining time was dedicated to explaining the process to, completion of the cognitive load scale (see Additional file 2) by, and rotation of, the students.

The context provided consisted of a paper-patient case. The relevant context focused on the patients with a musculoskeletal problem and the irrelevant context on patients with problems of the nervous system. Familiar context was context with which we thought the students were more able to identify with, as they were common diseases. Unfamiliar context were diseases that we thought students would have almost never heard off. See Table 2 for an overview of the learning tasks and the provided context in each group and Additional file 1 for examples of paper-patient cases and assignments.

**Table 2:** Overview of the learning tasks and the provided (ir) relevant and (un) familiar context^a^

Learning task	Group 1	Group 2	Group 3	Group 4	Group 5
	No context	Relevant-familiar context	Relevant-unfamiliar context	Irrelevant-familiar context	Irrelevant-unfamiliar context
Shoulder	Not applicable	Dislocated shoulder	Impingement of supraspinatus muscle	Parkinson’s disease	Hallevorden-Spatz disease
Wrist	Not applicable	Carpal tunnel syndrome	Trigger finger	Multiple Sclerosis	Huntington’s Disease
Knee	Not applicable	Ruptured knee ligaments	Patellofemoral pain syndrome	Alzheimer’s disease	Möbius syndrome
Ankle	Not applicable	Sprained ankle	Anterior compartment syndrome	Meningitis	Von Recklinghausen disease (neurofibromatosis)

The students completed a questionnaire assessing the participants’ familiarity with the context provided in the learning task to ascertain the chosen context were indeed (un) familiar to the students

^a^For clarity of reading, we have chosen to use the term ‘disease’ in this article when referring to the pathology/complaint/problem/affliction/condition/diagnosis of the musculoskeletal or neurological system on which the paper-patient was based

### Influence of fellow students, teachers & study material

Students worked individually and were not allowed speaking with each other, which was monitored by supervisors. There were no teachers available to explain the studied information. As many modern anatomy textbooks include clinical context that may interfere with the purposes of the context of the experiment, we provided the students with copied pages of the Sobotta Atlas of Human Anatomy [32] and Clinically Oriented Anatomy [33] that only contained anatomical information. All these precautions were taken to limit the influence of other factors besides the given context on students’ learning.

### Data analysis

Analysis of covariance (ANCOVA) was performed with posttest score (i.e. sum score of 0–40) as response variable. Relevance (i.e. relevant vs. irrelevant) and familiarity (i.e. familiar vs. unfamiliar) were included as dummy variables to test the hypotheses that ‘learning with a paper-patient context leads to better performance than learning without context’ (**H1**), ‘relevant context leads to better performance than irrelevant context’ (**H2**) and ‘familiar context leads to better performance than unfamiliar context’ (**H3**). The self-perceived learning score averaged over the four stations and was included to test the hypothesis that ‘higher scores on the self-perceived learning scale predict higher performance’ (**H4**). Pretest score (i.e. sum score of 0–40) was included as covariate to maximize statistical power through reduction of unexplained variance in posttest score. Mean centering was applied to self-perceived learning and pretest, so that the intercept reflects the expected performance in the control condition (i.e., irrelevant, unfamiliar, and no context) for average self-perceived learning and average pretest performance.

## Results

### Familiarity of context

The results of the students’ familiarity with the context showed an average score for the presumably familiar diseases of 2.11 (sd 0.69) for the musculoskeletal and 2.50 (sd 0.67) for the neurological subjects. The average score for the presumably unfamiliar diseases were 0.16 (sd 0.32) for the musculoskeletal and 0.33 (sd 0.26) for the neurological subjects. There was a significant difference between the familiar and unfamiliar context (*p* < .001), showing that students were indeed much more familiar with the diseases used in the patient cases in the ‘familiar context groups’. This increases the chance that these familiar contexts indeed activated prior knowledge in students, which was the aim.

### Context and performance

Cronbach’s *α* was 0.58 for the pretest and 0.76 for the post-test. A plausible explanation for the somewhat lower internal consistency of the pretest is a restriction-of-range effect in knowledge prior to the experiment; the contents covered in the experiment had not yet been offered in the students’ curriculum at that point in time.

Table 3 presents the results of the ANCOVA for performance as response variable. No multicollinearity problems were encountered, tolerance values varied from 0.683 for context to 0.966 for self-perceived learning. Against expectations, the hypothesis that ‘learning with a paper-patient context leads to better performance than learning without context’ (**H1**) could not be confirmed. Results even show an opposite effect (*β* = −0.301). The hypothesis that ‘relevant context leads to better performance than irrelevant context’ (**H2**) is not supported convincingly, and the standardized beta (*β* = 0.091) indicates a small effect (in the expected direction) at best. As expected, ‘familiar context leads to better performance than unfamiliar context’ (**H3**) (*p* < .05). The standardized beta (*β* = 0.169) indicates that the effect of familiarity is in the range of small (0.10) to medium (0.25) [34].

**Table 3:** ANCOVA for the effects of context relevance and context familiarity on posttest performance (H1-H4)

Effect	*B* (SE)	*β*	t (139)	*p*-value	95 % confidence interval	
					Lower	Upper
Intercept	20.015 (0.768)		26.061	< 0.001	18.497	21.534
Context^a^	-3.803 (1.027)	-0.301	-3.704	< 0.001	-5.834	-1.773
Relevant^b^	0.942 (0.784)	0.091	1.201	0.232	-0.608	2.492
Familiar^c^	1.769 (0.789)	0.169	2.244	0.026	0.210	3.329
Self-perceived learning^d^	1.018 (0.354)	0.196	2.874	0.005	0.318	1.719
Pretest^e^	0.830 (0.113)	0.507	7.333	< 0.001	0.606	1.054

*β*-values around 0.10, 0.25 and 0.40 are indicative of small, medium, and large effects, respectively

^a^context (1) vs. no context (0); Hypothesis 1 ‘learning with a paper-patient context leads to better performance than learning without context’ could not be confirmed

^b^relevant (1) vs. irrelevant (0); Hypothesis 2 ‘relevant context leads to better performance than irrelevant context’ is not supported convincingly

^c^familiar (1) vs. unfamiliar (0); Hypothesis 3 ‘familiar context leads to better performance than unfamiliar context’ is confirmed

^d^mean centered; Hypothesis 4 ‘higher scores on the self-perceived learning scale predict higher performance’ is confirmed

^e^mean centered; Even in the pretest, participants scored significantly better on the test questions with context than on test questions without context

In both the pre- and posttest, participants scored significantly better on the test questions with context than on test questions without context (pretest 5.04 (±1.95) vs 2.91 (±1.48) points (*p* < .001); posttest 9.50 (±2.74) vs 8,61 (±3.01) points (*p* < .001) out of 20). The treatment condition did not influence students’ performance on test questions with and without context.

### Context and cognitive load

The scores of intrinsic and extraneous cognitive load and self-perceived learning is an average of the scores measured after each station in the learning task. In the original paper by Leppink and colleagues (2013), the self-perceived learning (then: ‘germane cognitive load’) score was the average of four items. In the current study, one of the four items had a considerably, but explainable (see Additional file 2), lower item-total correlation, lowering the internal consistency of the scale (Cronbach’s *α* of the original four-item scale ranged from 0.50 to 0.66). Therefore, the average of the remaining three items was taken as self-perceived learning score in the current study (Cronbach’s *α* of this revised scale ranged from 0.77 to 0.84).

The absence of context, or the presence of (ir) relevant-(un) familiar context does not influence the scores on the cognitive load and self-perceived learning subscales. Nonetheless, independent of treatment condition, ‘higher scores on the self-perceived learning scale predict higher performance’ (**H4**) as expected, see Table 3. The standardized betas (*β*) indicate that the effect of self-perceived learning is in the range of small (0.10) to medium (0.25) [34].

## Discussion

### Influence of context on performance

We investigated the influence of providing a paper-patient case as a context for learning, as the literature suggested that this context would improve learning though the mechanisms associated with the cognitive (semantic) dimension of context. Our results surprisingly show that students who studied the content (anatomical knowledge) with context did not perform better (and in some cases even worse) than students who studied the content without context. It is possible that context as operated in this study (being a paper-patient case) has minimal impact on the type of learning (anatomical knowledge) that occurred. This finding may potentially be explained by an interaction of the expertise level of the participants, the nature of anatomical knowledge and approaches to learning by students.

It is generally known that one of the most important factors affecting learning is what a learner already knows, or in other words, a learners’ prior knowledge is the most important pre-requisite for learning [13, 35, 36]. The expertise level regarding anatomy of the musculoskeletal system of the complete group of participants was low, being first year medical students who had not studied this region before entering in this study (average score on pretest was 8.1 points out of 40); this low expertise level may have influenced their approach to learning during the learning task.

Our understanding of how students approach learning is informed by the research of Marton and Säljö [37, 38], whose results showed that students’ approaches to study could be divided into two categories: surface and deep. A surface approach to learning is associated with an intention to memorize information (facts) in isolation and recite them back in examinations. It invites routine memorization, ‘rote-learning’ or ‘mechanical repetition’, of facts and lists, often aided by the use of mnemonics. A deep approach to learning is characterized by a motivation to understand the topic. Students using a deep approach try to make the information meaningful. While learning, students try to make connections between facts and with previous knowledge, seek structure within the material, search for principles and integrate facts across domains [39–42]. Students with a higher expertise level (more prior knowledge) have more ideas to which they can relate new information and so can more easily engage in such processes as meaningful learning and elaboration [36]. Therefore it is possible that the participants of this study used a surface approach to learning during the learning task.

Furthermore, anatomy is a discipline with its own language to describe the organization and structures of the body [40]. Students have described this large amount of vocabulary and facts as daunting, in contrast to for example learning physiological knowledge [43, 44]. It has also been previously reported that, in sciences where there is a complex vocabulary associated with learning, a deep approach may require a preliminary stage of rote learning that is difficult to distinguish from a surface approach [45]. Rote learning in this instance might be an example of an intention to understand, even though memorizing jargon or symbols is part of the learning process [46]. It is likely that the students spent the learning task rote-learning the names of the structures (surface approach), and did *not* engage in a deep approach by for example trying to understand how the anatomical content could explain signs and symptoms described in the paper-patient case of each station. Theoretically, the (characteristics of the) context in which content is learned may only have a positive effect on performance when students attempt a deep approach to learning. And with respect to anatomy, taking a deep approach to learning may only be possible when students have mastered a certain level of ‘anatomical vocabulary’ and thus have a higher expertise level. Our results even suggest that providing context while students are in a surface learning phase may work counterproductive. If both expertise level of students and the nature of anatomical knowledge indeed lead to a necessary primary stage of surface learning, and context may only influence learning while taking a deep approach, this may have a significant impact on the way the content is taught. Further research should therefore investigate these interactions.

## Influence of the relevance and familiarity of the context on performance

As described in the introduction, literature suggested that the mechanisms through with the cognitive dimension of context should improve learning and retrieval are activation of prior knowledge and storage of retrieval cues/pathways. We hypothesized that the characteristics of the context, relevance and familiarity, would influence these mechanisms. The characteristics of the context had indeed a small positive effect in the expected direction. Results of this study suggest that having a relevant-familiar context reduces the possible negative impact of being provided with a context.

Providing students with a context was thought to improve the quality of semantic networks by increasing the amount of information parts (retrieval cues) and increasing the amount of links (retrieval pathways). A high quality network should improve learning and retrieval of knowledge. To add to the literature on learning in context, we investigated whether a relevant context has increased the amount of retrieval cues and pathways more than irrelevant context and no context. Although a situation in which a student encounters an irrelevant context in an educational setting is improbable, it is reassuring that our results show a trend that a relevant context indeed leads to better performance.

Furthermore, providing students with a context was thought to activate prior knowledge (stored within semantic networks). A semantic network needs to be activated before meaningful connections between the prior knowledge in that network and the new information in the content and context can be made [13]. The results of this study seem to indicate that choosing a disease with which the students are most likely familiar is preferable above diseases with which they are unfamiliar. As semantic networks are idiosyncratic, it is probable that a familiar context activates prior knowledge in a larger group of students than an unfamiliar context. Furthermore, it is plausible that a familiar context (disease) will activate a much larger semantic network (more prior knowledge) about a subject than an unfamiliar context, because students will be better able to relate information in the context to what they already know. This is an important finding, because it is not unusual for teachers to introduce an uncommon disease to illustrate some basic science concept or principle. Our results suggest preventing those situations.

Even though the expertise level of the group of participants as a whole was low, the prior knowledge within the group varied widely (ranging between 3 and 19 points out of 40 on the pretest). The results show that scores on the pretest had a great effect on performance in the posttest, which seems to confirm that a learners’ prior knowledge is the most important pre-requisite for learning [13, 35, 36]. Activation of prior knowledge by a familiar context seems therefore very important. There was no interaction between the scores on the pre-test and the treatment condition in which the student performed the learning task. This indicates that there was no expertise-reversal effect: students with more prior knowledge of one of the musculoskeletal subjects did not benefit less from the content being presented in context than students with less prior knowledge. Providing a familiar context might therefore benefit all students.

Since the present study is the first to investigate the relationship between the characteristics of a context (relevance and familiarity), the mechanisms through which the cognitive dimension of context is said to improve learning (activation of prior knowledge, increasing retrieval cues/pathways), and test performance (increased acquisition and recall of knowledge), more research is necessary to further investigate and explain our findings.

### Limits of the study

Some aspects of the experimental design might have influenced the results discussed above. First, all groups were given the same amount of time during the learning task to study the content. The students in the ‘without context’ treatment condition did not have a patient case to read through, so they could invest all the available time on studying the content. It is possible that this increased ‘time on task’ contributed to a better than expected performance on the post-test. It was debated to give students in the without context condition a completely different text to read (for example a fairytale). However, as described in the introduction, context does not need to be relevant to the to-be-learned content to help create retrieval cues. Therefore we opted for a fully ‘without context’ condition. Furthermore, a patient case had on average 364 words, which takes such a short time to read we assume that there is a limited impact on time on task.

A further limitation of this study could be that students only studied the content *in context*, but did not apply the learned content *to context*. For example, an X-ray was included in the patient case about the dislocated shoulder, but students were *not* asked to identify the bones (e.g. scapula, humerus, clavicle) and/or bone markings (e.g. acromion, coracoid process, greater tubercle) which they studied in the to-be-learned content on the X-ray. In other words, students were not asked to actively make connections between content and context, or explain for example signs or symptoms in the context with the content.

A total of 158 students completed the pretest, but 145 students participated in the learning task and completed the posttest. Analysis showed that the majority of students that have withdrawn emanated from group 4 and that their pretest scores were lower than average. Subsequently, only students who scored above average studied the content in the irrelevant-familiar context and completed the posttest. Therefore a limitation of the study is that the effect of relevance could now be slightly underestimated and the effect of familiarity slightly overestimated.

The last limitation is that the results would have had more power if it was possible to calculate interactions. However the experimental design could not reach a full 3-way factorial (2x2x2) design, as it proved difficult to come up with a solution on how to create a learning task ‘*without* (ir) relevant and (un) familiar context’, or in other words how to manipulate the relevance and familiarity of the context when the context is absent. A full 2x2x2 design would also offer the possibility of comparing groups with the same amount of participants, instead of the comparison of the control condition to all other conditions as done in this study.

## Influence of context on cognitive load and knowledge retrieval

As predicted, the results showed that higher scores on the self-perceived learning scale, indicating that the student found the instructional features beneficial for learning, predict higher performance. However, one might expect that students with an irrelevant context would score significantly higher on the extraneous cognitive load scale, as irrelevant context could be seen as instructional features that are not beneficial for learning. An explanation could be the manifestation of a ‘nonsense effect’: students avoided engagement with the context because the patient case did not make sense to them in combination with the content that needed to be learned. Consequently, the instructional features of the irrelevant context would not have added to the students (extraneous) cognitive load.

Taking into account the theory of Transfer-Appropriate Processing, half of the test questions were formulated with context (patient vignette) and half of the test questions were formulated without context. Interestingly, the results show that students performed significantly better on the questions with context, not only in the posttest but already in the pretest! Whether the students studied the content with or without context did not influence performance on the different test questions. This contradicts the findings of Prince et al. [47], who showed that student taught in a clinical context performed better on a subtest with clinical context. The 40 test questions, 10 of each of the 4 musculoskeletal region, were presented in a mixed order. However, the option lists from which they had to pick an answer would contain all the muscles, bone (markings) or ligaments of all regions. Context may have had a ‘framing effect’: psychological research has shown that the way a question is ‘framed’ (the words used) influences how people answer the question [48]. In this study, the context within the question may stimulate activation of an appropriate semantic network and therefore increase the chance students’ pick the correct answer out of the option list. The results make it tentative to state that context aids retrieval (or improve correct guessing) of (anatomical) knowledge for (first year medical) students, but not the acquisition of knowledge as much as expected. However, the study design does not allow any conclusions to be drawn in this direction and it may be interesting to pursue these effects in further studies. Especially since results of a study investigating the difference between short case and factual knowledge questions in problem-solving assessment has shown that placing the questions in a realistic context has a considerable effect on the type of cognitive operations (thinking processes) which take place [49].

## Conclusions

The relationship between context and learning is much more complex than originally expected. The study of Koens et al. [50], which focused on the physical dimensions of context, the study of Böckers et al. [39], which focused more on the affective dimension of context, and the present study focusing on the cognitive dimension of context all failed to find a significant positive influence of learning in a clinically relevant context. Our results suggest that relevance and familiarity of a paper-patient case positively influence the contribution of the cognitive dimension of context on increased acquisition and recall of knowledge. However, an interaction with expertise level and approaches to learning may exist. Experiments should systematically vary prior knowledge, familiarity, learning approach and cognitive load and search for consistent interactions. We may subsequently refine our theories around the role of context in learning.

## Additional files

Additional file 1: **Excerpt of two student manuals. These excerpts contain part of the learning task of the shoulder region (content) with a relevant-familiar and irrelevant-familiar patient case (context).** The manual of the students in the ‘without context’ treatment condition only contained the assignments and no patient case. For a full overview of the learning tasks and the diseases on which the patient cases in the (ir)relevant-(un)familiar treatment conditions were based, see Table 2. (DOC 47 kb) Additional file 2: **The cognitive load and self-perceived learning questionnaire of Leppink et al.** **[**27, 28**]** **tailored to the domain of anatomy education.** (DOC 30 kb)
