# Supplementary material for: Effects of learning content in context on knowledge acquisition and recall: a pretest-posttest control group design
Source: BMC Med Educ. 2015 Aug 15;15:133. doi: 10.1186/s12909-015-0416-0 (PMC4542121; doi:10.1186/s12909-015-0416-0)
Supplement: Additional file 1: — Excerpt of two student manuals. These excerpts contain part of the learning task of the shoulder region (content) with a relevant-familiar and irrelevant-familiar patient case (context). The manual of the students in the ‘without context’ treatment condition only contained the assignments and no patient case. For a full overview of the learning tasks and the diseases on which the patient cases in the (ir)relevant-(un)familiar treatment conditions were based, see Table 2. (DOC 47 kb) [file 12909_2015_416_MOESM1_ESM.doc]

**APPENDIX 2**

Excerpt of two student manuals. These excerpts contain part of the learning task of the shoulder region (content) with a relevant-familiar and irrelevant-familiar patient case (context). The manual of the students in the ‘without context’ treatment condition only contained the assignments and no patient case. For a full overview of the learning tasks and the diseases on which the patient cases in the (ir)relevant-(un)familiar treatment conditions were based, see table 2.

| Excerpt of student manual of the shoulder station in the relevant-familiar treatment condition (dislocated shoulder)* | Excerpt of student manual of the shoulder station in the irrelevant-familiar treatment condition (Parkinson’s disease)* |
| --- | --- |
| A gymnastics teacher enters the emergency room with Ben, 17 years old, who had a nasty fall while performing a jump over the vaulting table. Ben landed on his outstretched arm. He supports his forearm while walking and sitting. Ben’s shoulder hurts a lot and he cannot move his arm, therefore you need to cut his T-shirt in order to perform the physical examination. Upon inspection, you can see a clear difference between the two shoulders (figure X). You tell Ben that you think his shoulder is dislocated. After consultation with your supervisor you decide to have an X-ray taken (figure X). The X-ray confirms your diagnosis: an anterior dislocation of the head of the humerus.  **Assignment 1**  The shoulder joint, or ***glenohumeral joint***, has two articular surfaces. The first articular surface is formed by the ***glenoid cavity*** of the ***scapula***. Study the following bone markings of the scapula:   - ***glenoid cavity*** - ***spine of the scapula*** - ***acromion*** - ***coracoid process*** - ***supraglenoid tubercle*** - ***infraglenoid tubercle***   Replacing an anterior dislocation can be done without anesthesia or a lot of pushing and shoving. There are two ways to place the head of the humerus back in the glenoid cavity: 1) the patient lies in prone position and a weight is attached to the arm or 2) by rotation of the shoulder (figure X). You decide to try the first technique with Ben. You explain to Ben that the muscles around the shoulder will eventually get tired by the pull of the weight, and then the head of the humerus automatically slides back in place. At the moment that happens, Ben will immediately have much less pain.  **Assignment 2**  The second articular surface of the glenohumeral joint is formed by the head of the humerus. Study the following bone markings of the ***humerus***:   - ***head of the humerus*** - ***greater tubercle*** - ***lesser tubercle*** - ***intertubercular sulcus (bicipital groove)*** - ***lateral epicondyle*** - ***medial epicondyle***   **Assignment 3**  Within the glenohumeral joint you will find one of the tendons of the ***biceps brachii muscle***. This is unique to the glenohumeral joint; you will not find tendons in any other joint of the human body. The biceps brachii muscle consists of two heads: the ***long head*** and the ***short head***. These two heads merge into one tendon that attaches to the ***radial tuberosity*** of the ***radius*** (one of the bones in the forearm). What are the names of the bone markings of the scapula to which the tendons of the ***long head*** and ***short head*** attach (see assignment 1)?   | **Long head** |  | | --- | --- | | **Short head** |  |   The mother of Ben comes to the hospital to pick him up. She is afraid that Ben’s shoulder will dislocate more often now it happened ones. She asks you whether Ben should be extra careful in the future. You tell her it is possible that his shoulder dislocates again, but that he could train the muscles around his shoulder to reduce the risk.  The glenohumeral joint has a joint capsule which is slack to provide for a lot of movement (figure X). Possible movements in the glenohumeral joint are ***abduction & adduction, anteflexion & retroflexion*** and ***endorotation and exorotation*** (figure X). Normally there are 4 muscles that help to prevent dislocation of the shoulder during these movements. Together these muscles are called the ‘rotatorcuff’.  **Assignment 4**  The 4 muscles of the rotatorcuff are:   - supraspinatus muscle - infraspinatus muscle - teres minor muscle - subscapularis muscle   Study the position of these muscles. To which bony markings of the humerus do each of these muscles attach (see assignment 2)?   | **supraspinatus** |  | | --- | --- | | **infraspinatus** |  | | **teres minor** |  | | **subscapularis** |  | | You are an intern in the practice of a general physician. When you pick up Ed, 69 years old, from the waiting room, you notice he has trouble to start walking and walks with small steps. While he speaks with the GP you see a resting tremor: a constant movement of Ed’s hand and fingers, as if he is a rolling a cigarette rolling. Ed’s face shows almost no expression. His speech is monotonous, but there is nothing wrong with him intellectually. Adding it all you recognize hypokinetic (lack of movement) image of a patient with Parkinson's disease (figure X).  **Assignment 1**  The shoulder joint, or ***glenohumeral joint***, has two articular surfaces. The first articular surface is formed by the ***glenoid cavity*** of the ***scapula***. Study the following bone markings of the scapula:   - ***glenoid cavity*** - ***spine of the scapula*** - ***acromion*** - ***coracoid process*** - ***supraglenoid tubercle*** - ***infraglenoid tubercle***   The cause of Parkinson's disease is degeneration (necrosis) of the nerve cells in the basal ganglia. The basal ganglia consist of the caudate nucleus, putamen, globus pallidus, subthalamic nucleus and the substantia nigra. They surround the thalamus with exception of the substantia nigra which is located in the brain stem. Together they are involved in the control of movements. Parkinson's disease is primarily a result of degeneration of the substantia nigra (figure X), which results in the decrease of the formation of the neurotransmitter ‘dopamine’. As a result the thalamus and the other basal ganglia will dysfunction.  **Assignment 2**  The second articular surface of the glenohumeral joint is formed by the head of the humerus. Study the following bone markings of the ***humerus***:   - ***head of the humerus*** - ***greater tubercle*** - ***lesser tubercle*** - ***intertubercular sulcus (bicipital groove)*** - ***lateral epicondyle*** - ***medial epicondyle***   **Assignment 3**  Within the glenohumeral joint you will find one of the tendons of the ***biceps brachii muscle***. This is unique to the glenohumeral joint; you will not find tendons in any other joint of the human body. The biceps brachii muscle consists of two heads: the ***long head*** and the ***short head***. These two heads merge into one tendon that attaches to the ***radial tuberosity*** of the ***radius*** (one of the bones in the forearm). What are the names of the bone markings of the scapula to which the tendons of the ***long head*** and ***short head*** attach (see assignment 1)?   | **Long head** |  | | --- | --- | | **Short head** |  |   The medication for Parkinson's disease is called Levodopa, a synthesized form of dopamine which can easily cross the blood-brain barrier. Levodopa can replace dopamine, slowing down (but unfortunately not stopping) the worsening of the symptoms. In the past decade, ‘deep-brain stimulation’ has been applied on an experimental basis. The cells in the basal ganglia are stimulated using an electrode that is surgically implanted in the brain. When this electrode is activated, the patient’s tremors – quite spectacularly – disappear acutely.  The glenohumeral joint has a joint capsule which is slack to provide for a lot of movement (figure X). Possible movements in the glenohumeral joint are ***abduction & adduction, anteflexion & retroflexion*** and ***endorotation and exorotation*** (figure X). Normally there are 4 muscles that help to prevent dislocation of the shoulder during these movements. Together these muscles are called the ‘rotatorcuff’.  **Assignment 4**  The 4 muscles of the rotatorcuff are:   - supraspinatus muscle - infraspinatus muscle - teres minor muscle - subscapularis muscle   Study the position of these muscles. To which bony markings of the humerus do each of these muscles attach (see assignment 2)?   | **supraspinatus** |  | | --- | --- | | **infraspinatus** |  | | **teres minor** |  | | **subscapularis** |  | |
| * The mentioned figures are not included to make the excerpts more concise | |
