# Supplementary material for: Effects of learning content in context on knowledge acquisition and recall: a pretest-posttest control group design
Source: BMC Med Educ. 2015 Aug 15;15:133. doi: 10.1186/s12909-015-0416-0 (PMC4542121; doi:10.1186/s12909-015-0416-0)
Supplement: Additional file 2: — The cognitive load and self-perceived learning questionnaire of Leppink et al. [27, 28] tailored to the domain of anatomy education. (DOC 30 kb) [file 12909_2015_416_MOESM2_ESM.doc]

**APPENDIX 1**

The cognitive load and self-perceived learning questionnaire of Leppink et al. tailored to the domain of anatomy education

| All of the following 10 questions refer to the task (the station) that you just finished. Please take your time to read each of the questions carefully and respond to each of the questions on the presented scale from 0 to 10, in which ‘0’ indicates not at all the case and ‘10’ indicates completely the case.  (I.) The images and prosections in this station were very complex.  0 1 2 3 4 5 6 7 8 9 10  (II.) The anatomy covered in this station was very complex.  0 1 2 3 4 5 6 7 8 9 10  (III.) In this station, very complex terms were mentioned.  0 1 2 3 4 5 6 7 8 9 10  (IV.) The explanations and instructions in this station were very unclear.  0 1 2 3 4 5 6 7 8 9 10  (V.) The explanations and instructions in this station were full of unclear language.  0 1 2 3 4 5 6 7 8 9 10  (VI.) The explanations and instructions in this station were, in terms of learning, very ineffective.  0 1 2 3 4 5 6 7 8 9 10  (VII.) This station really improved my understanding of the anatomy that was covered.  0 1 2 3 4 5 6 7 8 9 10  (VIII.) This station really improved my understanding of the diseases that were covered.  0 1 2 3 4 5 6 7 8 9 10  (IX.) This station really improved my knowledge of the terms that were mentioned.  0 1 2 3 4 5 6 7 8 9 10  (X.) This station really improved my knowledge and understanding of anatomy in general.  0 1 2 3 4 5 6 7 8 9 10 |
| --- |

Questions 1, 2 & 3 measure intrinsic load, questions 4, 5 & 6 measure extraneous load and questions 7, 8, 9 & 10 measure self-perceived learning. The scores of each of the three factors is an average of the scores measured after each station in the learning task.

For calculation of self-perceived learning in this study, item VIII was excluded. That this item lowered the internal consistency of the scale is not surprising considering the fact that within the patient cases, only the signs and symptoms of a disease were described, added with short information on diagnosis and treatment. The pathophysiology of a disease was not extensively explained, which could consequently not have led to a great improvement of the understanding of the disease itself.
